# Supplementary material for: Precisely translating computed tomography diagnosis accuracy into therapeutic intervention by a carbon-iodine conjugated polymer
Source: Nat Commun. 2022 May 12;13:2625. doi: 10.1038/s41467-022-30263-1 (PMC9098856; doi:10.1038/s41467-022-30263-1)
Supplement: Supplementary file 1 — Supplementary Information [file 41467_2022_30263_MOESM1_ESM.pdf]

# Supplementary Information

## Precisely Translating Computed Tomography Diagnosis Accuracy into Therapeutic Intervention by a Carbon-Iodine Conjugated Polymer

*Mingming Yin[1], Xiaoming Liu[2,3], Ziqiao Lei[2,3], Yuting Gao[4], Jiacheng Liu[2,3], Sidan Tian[1], Zhiwen Liang[5], Ye Wang[5], Fanling Meng[1,6,7], Liang Luo[1,6,7]**

[1]National Engineering Research Center for Nanomedicine, College of Life Science and Technology, Huazhong University of Science and Technology, Wuhan 430074, China.

[2]Department of Radiology, Union Hospital, Tongji Medical College, Huazhong University of Science and Technology, Wuhan 430022, China.

[3]Hubei Province Key Laboratory of Molecular Imaging, Wuhan 430022, China.

[4]Faculty of Materials Science and Chemistry, China University of Geosciences, Wuhan 430074, China.

[5]Cancer Center, Union Hospital, Tongji Medical College, Huazhong University of Science and Technology, Wuhan 430022, China.

[6]Key Laboratory of Molecular Biophysics of the Ministry of Education, College of Life Science and Technology, Huazhong University of Science and Technology, Wuhan, 430074, China

[7]Hubei Key Laboratory of Bioinorganic Chemistry and Materia Medica, School of Chemistry and Chemical Engineering, Huazhong University of Science and Technology, Wuhan 430074, China.

*Email: liangluo@hust.edu.cn;

**Materials and Instruments**

1,4-Bis(trimethylsilyl)-1,3-butadiyne, silver nitrate, 2-(pyridin-3-yl)ethan-1-amine, and triethylamine were purchased from Energy Chemical. *N*-iodosuccinimide was purchased from J&K Scientific. Poly(maleic anhydride-alt-1-octadecene) was purchased from Sigma-Aldrich. Poly(ethylene glycol) amine (PEG-$NH_2$, molecular weight 5K) was purchased from SINOPEG. Iohexol ([I]: 300 mg $mL^{-1}$) was purchased from Ge Healthcare Shanghai Co., Ltd. Au fiducial markers were provided by Union Hospital, Wuhan, as a kind gift. Diethyl oxalate and all other reagents were purchased from Sinopharm Chemical Reagent Co. Ltd. All chemicals were used as received without further purification.

All NMR spectra were acquired on an Agilent 400-MR 400 MHz spectrometer, and chemicals shifts were referenced to the residue solvent peaks and given in ppm. X-ray single crystal diffraction was recorded on an XtaLAB PRO MM007HF C Single Crystal X-ray Diffractometer. Absorption spectra were acquired on a TU-1810DSPC UV/Vis spectrophotometer (Puxi General Instrumental Company, China) or a microplate reader (Varioskan LUX, Thermo Scientific, USA). TEM experiments were performed using a transmission electron microscope (Hitachi HT7700). Raman spectroscopy was performed using a LabRAM HR800 Raman spectrometer coupled with an infinity-corrected, confocal design microscope (Horiba Jobin Yvon), and a 785-nm laser was applied to the sample with 5 mW laser power. Elemental analysis was performed by energy-dispersive X-ray analysis of electron probe X-ray microanalyzer (EPMA-8050G). SEM experiments were performed in Nova NanoSEM 450. All CT scans were performed with a multi-detector CT scanner (SOMATOM Force, Siemens Healthcare, Erlangen, Germany). All SBRT data was collected

in CyberKnife® Robotic Radiosurgery System (Accuray Inc, Sunnyvale, CA, USA).

## Supplementary Figures

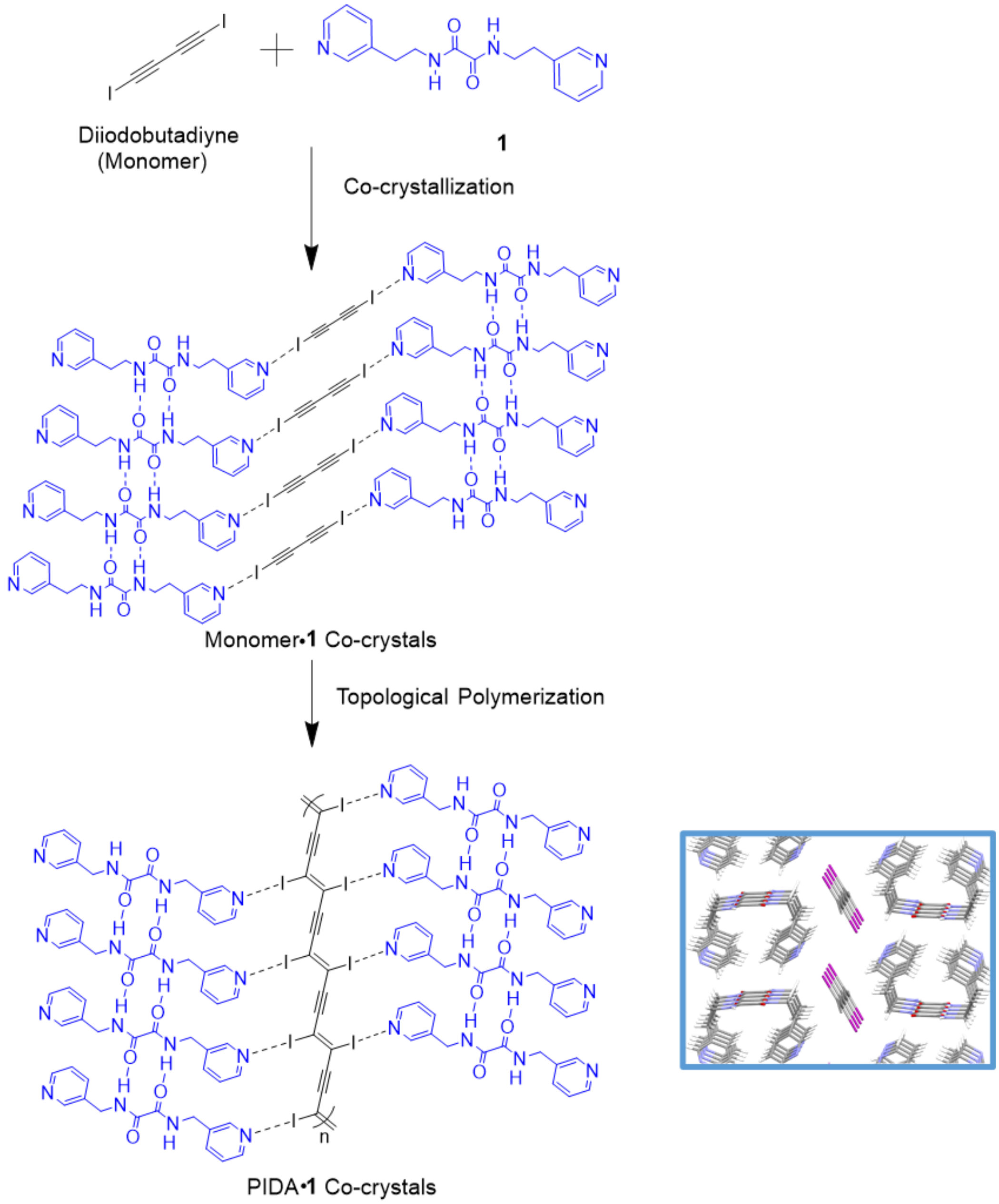


**Supplementary Fig. 1** Topological polymerization of diiodobutadiyne via the co-crystal strategy. Inset: Top view of the single-crystal structure of PIDA•**1** co-crystals. The unit cell parameters are a = 4.9544(3) Å, b = 28.1140(16) Å, c = 7.8721(5) Å, α = 90°, β = 95.277(3)°, γ = 90°, V = 1091.84 Å3, Z = 2.

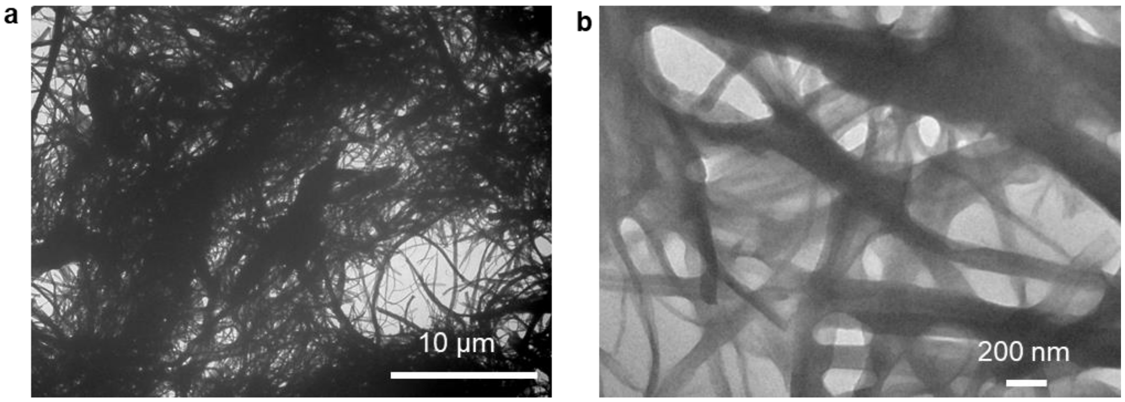


**Supplementary Fig. 2** TEM images of PIDA aggregates. The experiment was repeated 3 times independently with similar results.

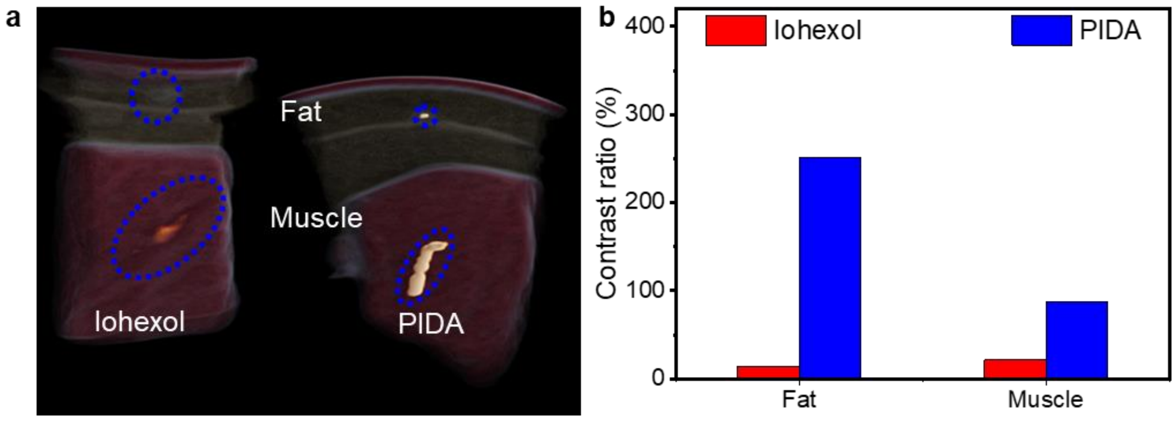


**Supplementary Fig. 3** PIDA-based ultraefficient CT imaging in vitro. **a** CT images of two porcine tissues injected with the PIDA suspension ([I]: 4 mg mL$^{-1}$) and iohexol solution ([I]: 4 mg mL$^{-1}$) respectively. **b** Contrast ratio of the injected PIDA and iohexol in porcine fat and muscle tissues. The contrast ratio was calculated as: $2\times(CT_{sample}-CT_{background})/(CT_{sample}+CT_{background})\times100\%$. Source data are provided as a Source Data file.

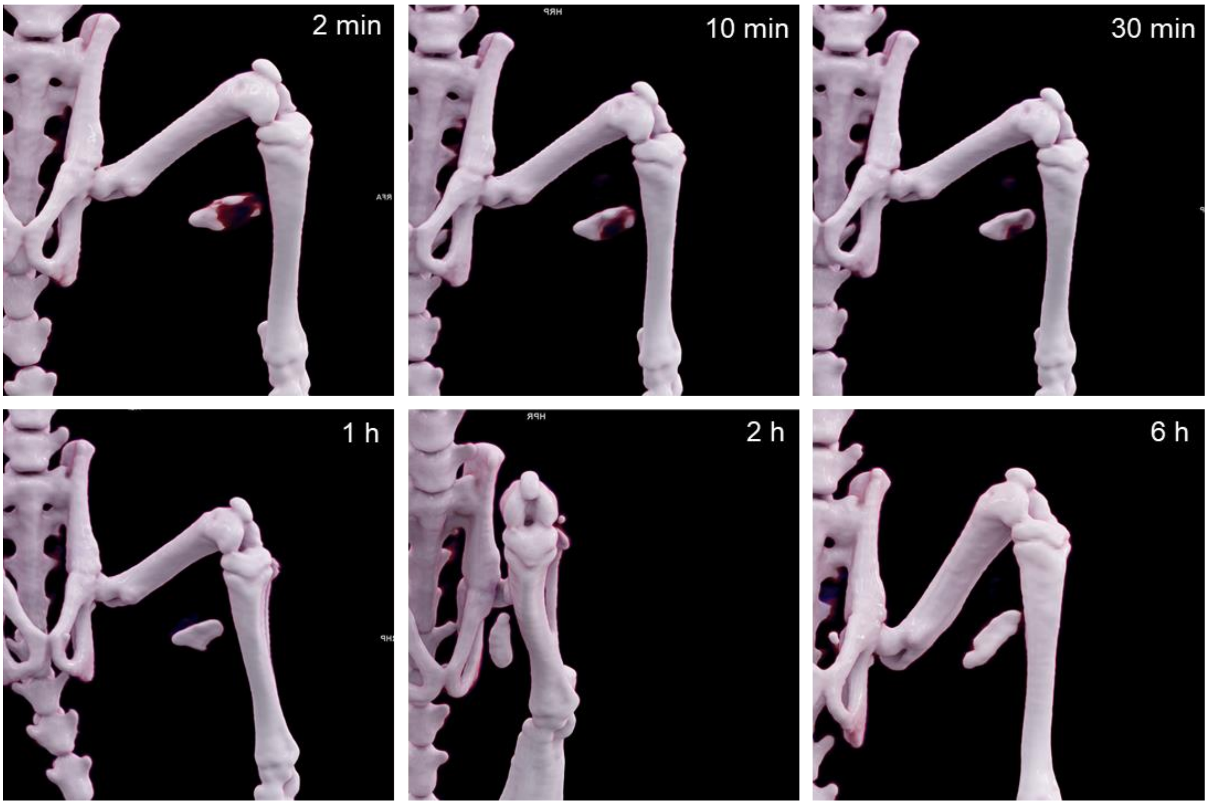


**Supplementary Fig. 4** CT images of a rat at different time after being injected with a PIDA suspension ([I]: 4 mg $mL^{-1}$).

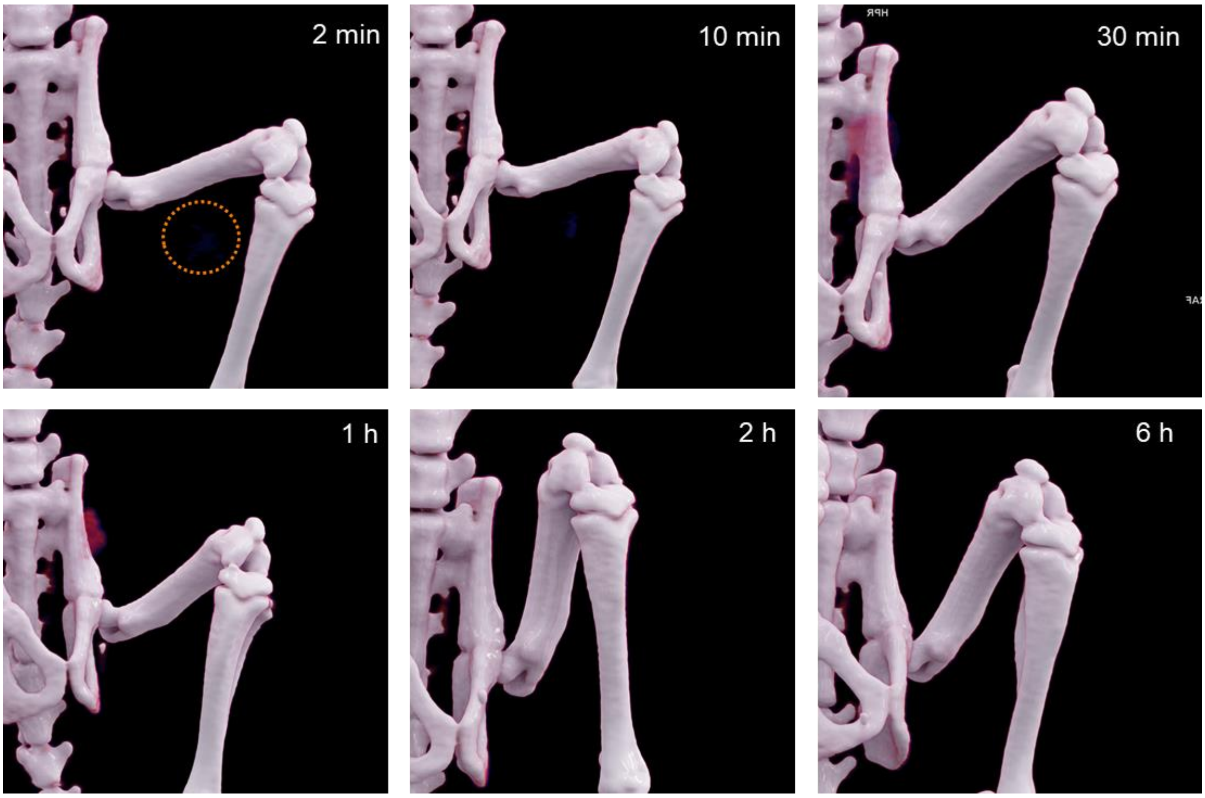


**Supplementary Fig. 5** CT images of a rat at different time after being injected with iohexol solution ([I]: 4 mg $mL^{-1}$).

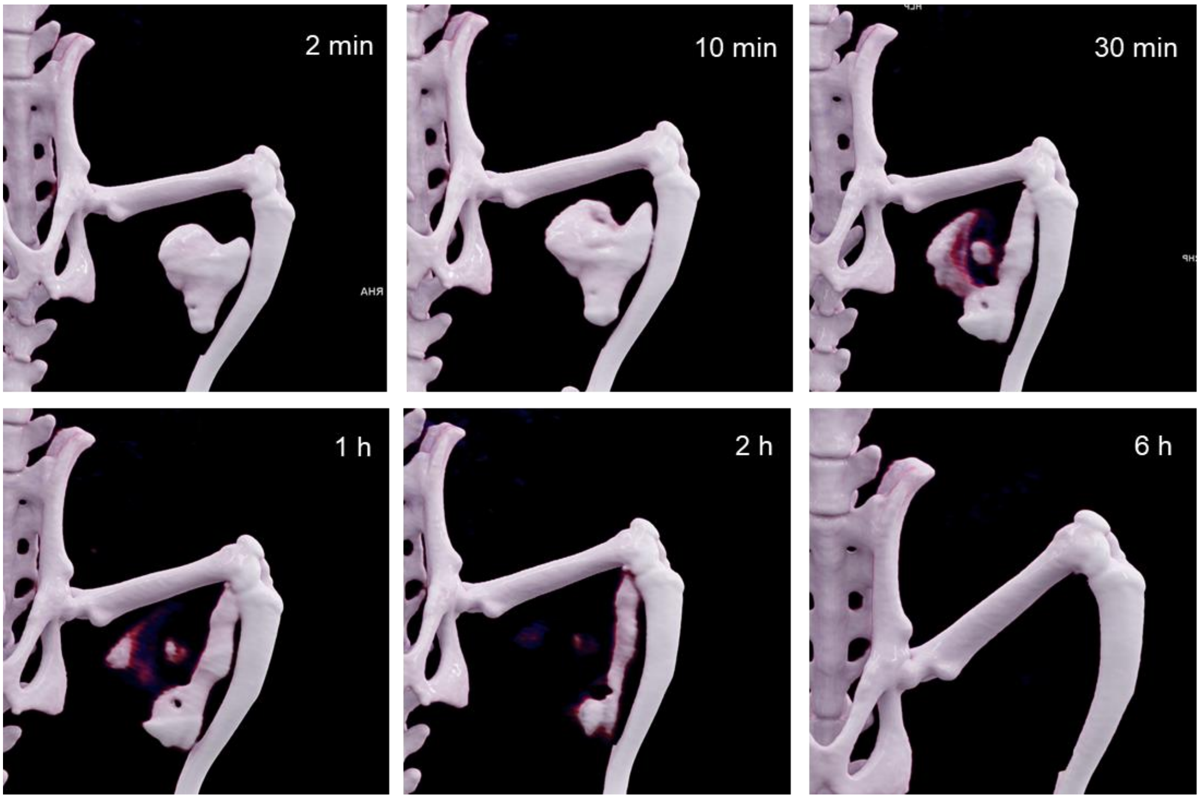


**Supplementary Fig. 6** CT images of a rat at different time after being injected with 200 μL iohexol ([I]: 100 mg $mL^{-1}$).

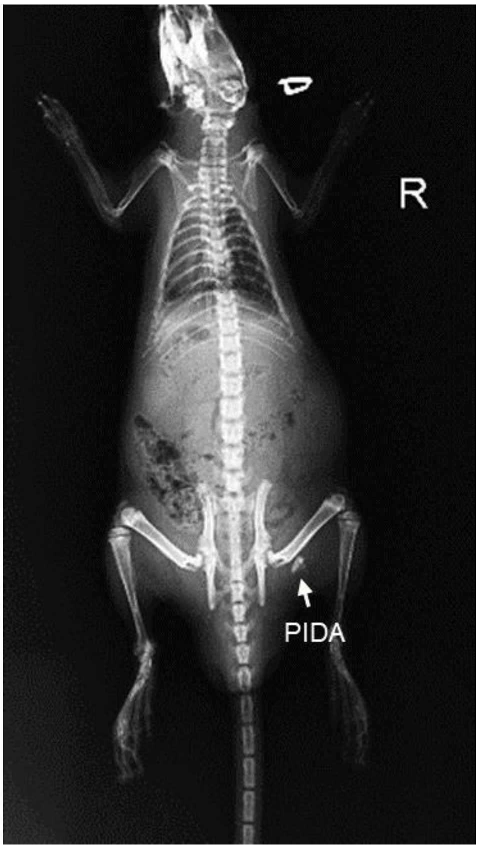


**Supplementary Fig. 7** X-ray image of the rat injected with 200 μL PIDA suspension ([I]: 4 mg $mL^{-1}$).

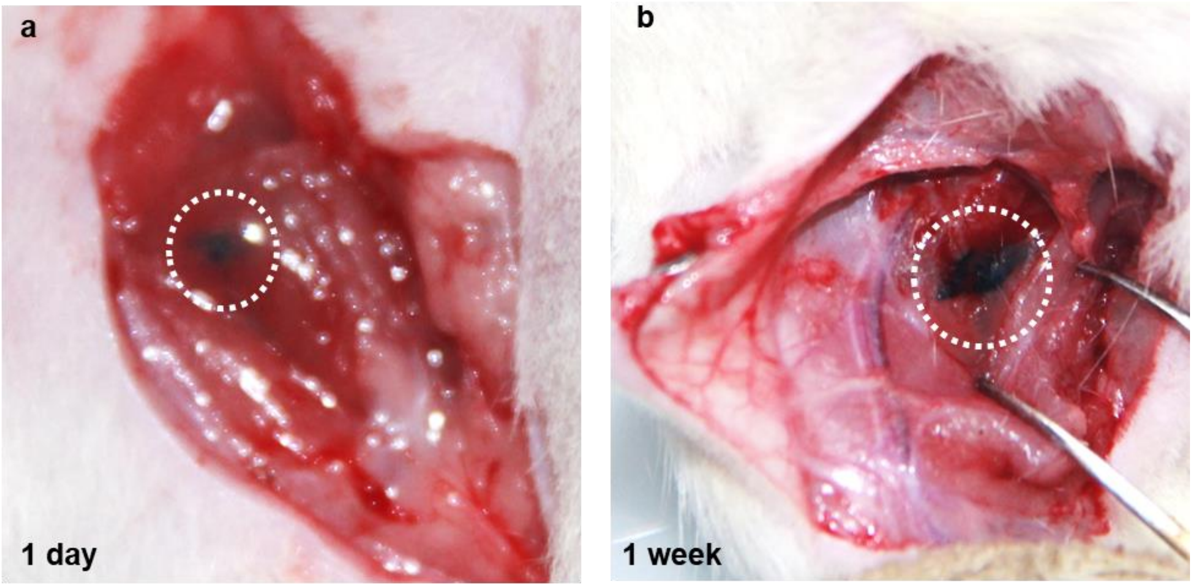


**Supplementary Fig. 8** Resected rat tissues after being injected with 200 μL of PIDA suspension ([I]: 4 mg mL$^{-1}$) for different time. **a** 1 day; **b** 1 week. The white circles indicated the deep blue color of injected PIDA.

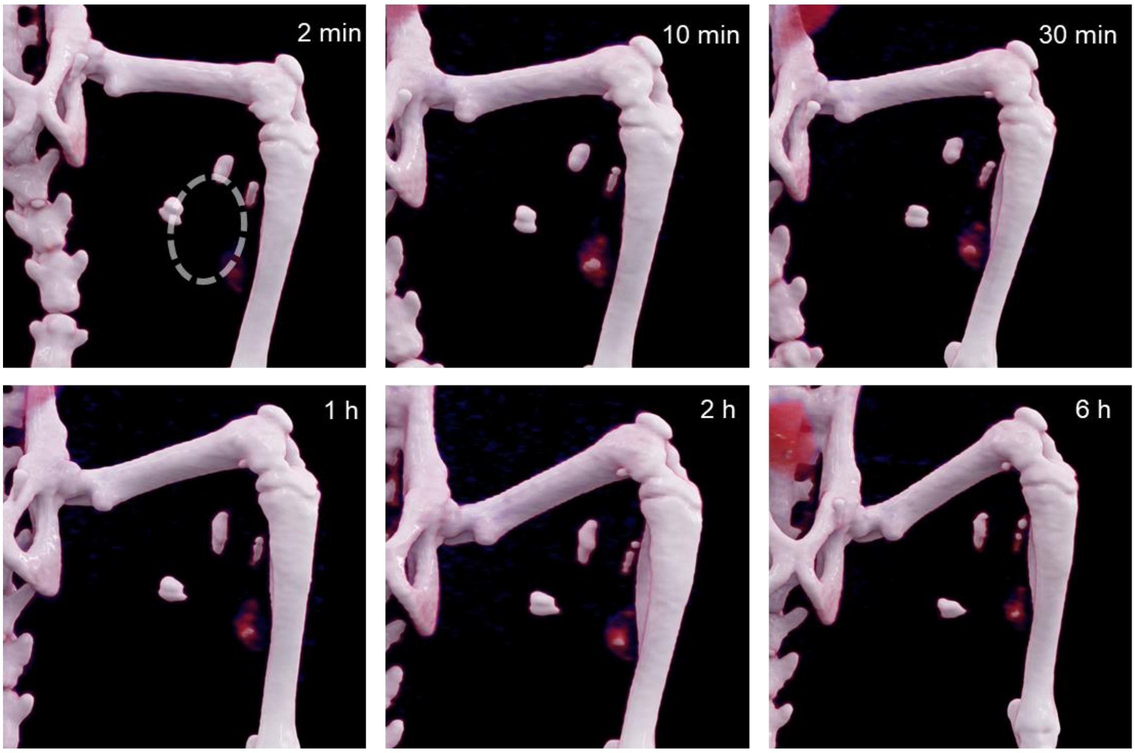


**Supplementary Fig. 9** CT images of a tumor-bearing rat injected with a PIDA suspension at 4 spots on the tumor periphery ([I]: 4 mg $mL^{-1}$, 50 μL each) over time. The white circle line indicated the position of the orthotopic xenograft tumor.

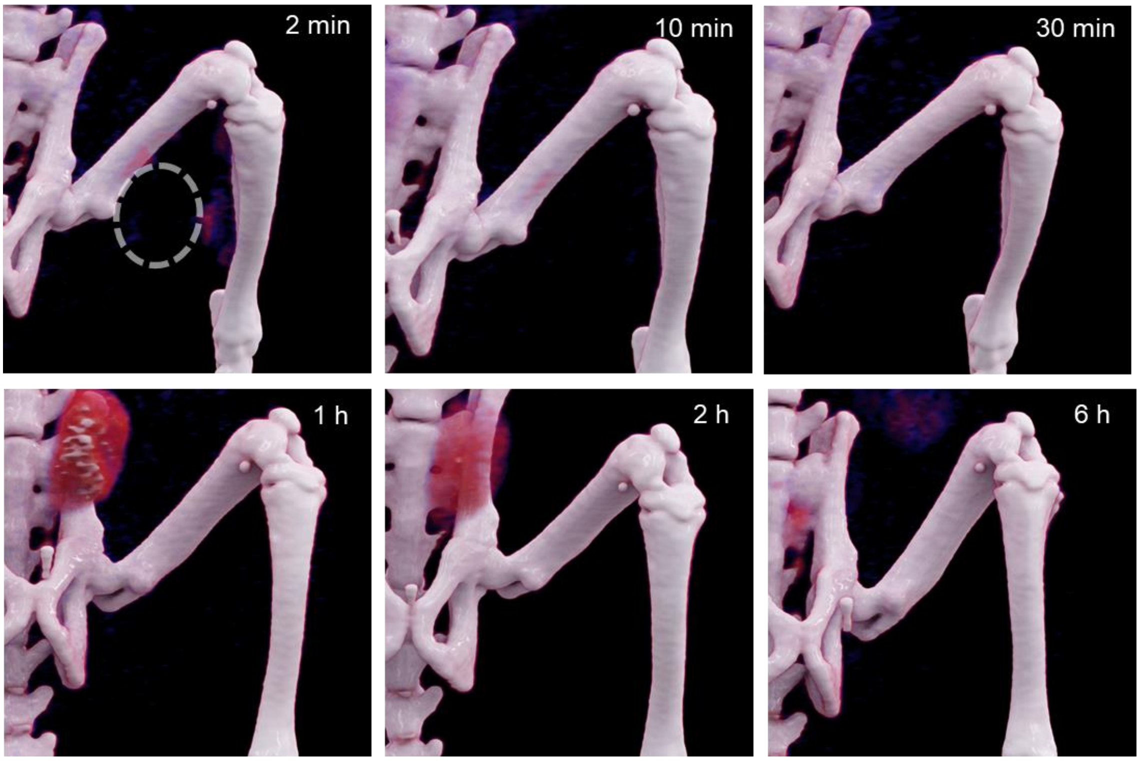


**Supplementary Fig. 10** CT images of a tumor-bearing rat injected with an iohexol solution at 4 spots on the tumor periphery ([I]: 4 mg $mL^{-1}$, 50 μL each) over time. The white circle line indicated the position of orthotopic xenograft tumor.

**Supplementary Table 1** Blood analysis of rats injected with different markers on Day 7. ALT: alanine aminotransferase, AST: aspartate aminotransferase, ALP: alkaline phosphatase, CERA: creatinine. The levels of AST and ASP of the rat with Au in the liver were higher than normal (shown in red).

| | Reference range | Au in liver | PIDA in liver | Control |
|---|---|---|---|---|
| ALT (U/L) | 33. 70～98. 70 | 97.8 | 59 | 68.2 |
| AST (U/L) | 69. 70～322. 90 | 406 | 192.2 | 191.5 |
| ALP (U/L) | 1. 30～211. 00 | 345 | 138 | 162 |
| CREA (U/L) | 19. 43～64. 97 | 29 | 17 | 17 |

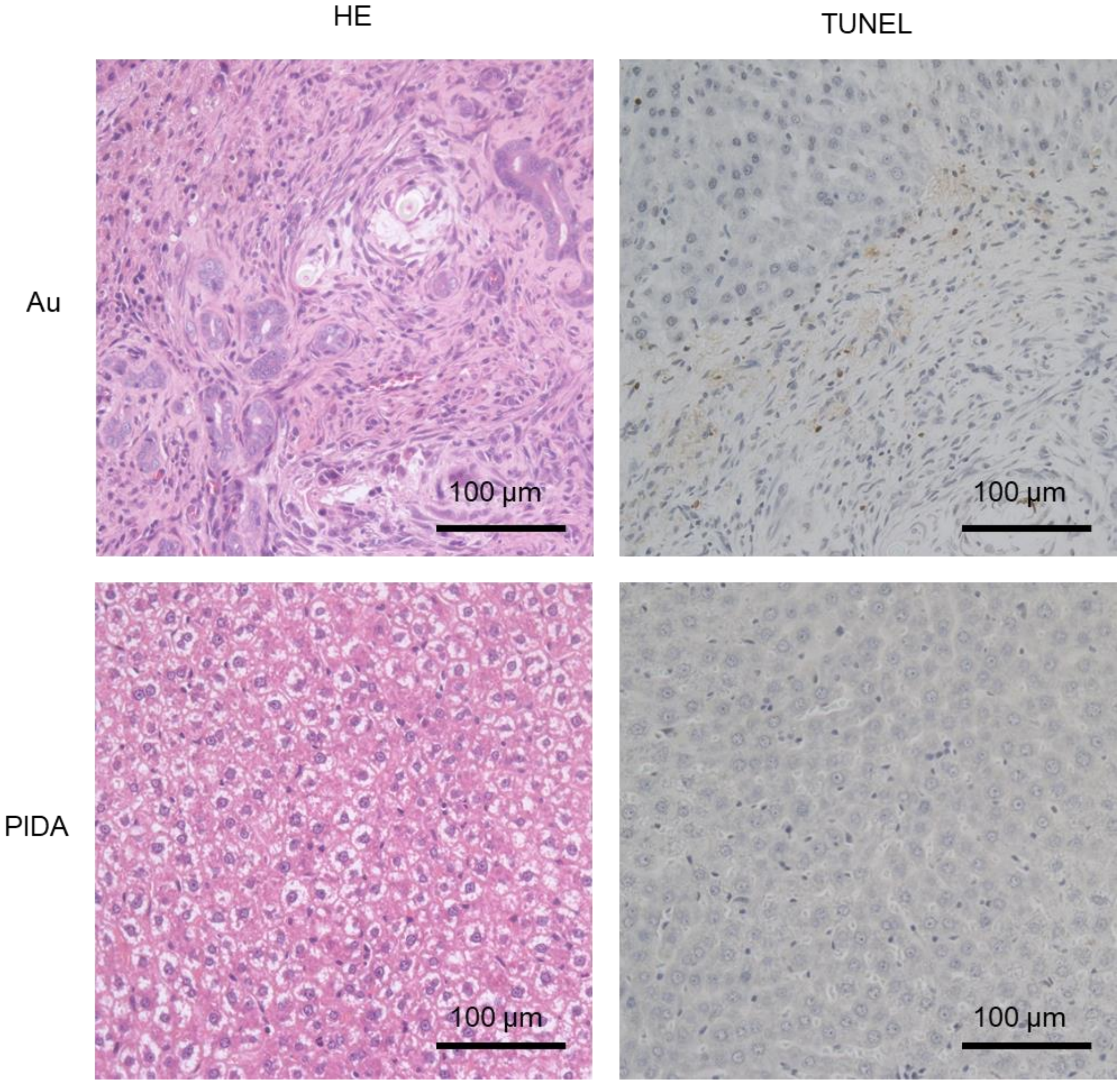


**Supplementary Fig. 11** Histologic section of the rat liver on Day 7. The H&E and TUNEL images indicated the damage of Au fiducial marker to liver tissue, while no abnormality was observed in the liver implanted with PIDA fiducial marker. The experiment was repeated 3 times independently with similar results.

a

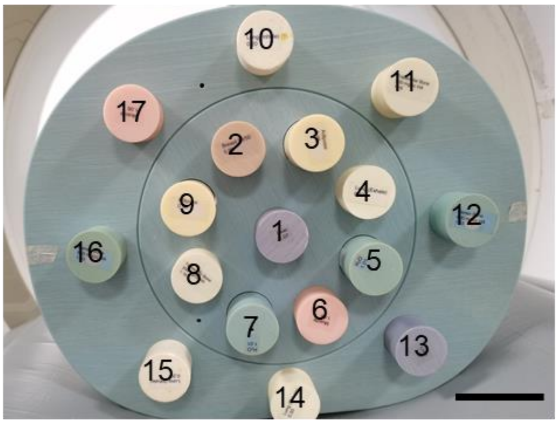


b

| | |
|---|---|
| 1&13 | Liver |
| 2 | Breast |
| 3&9 | Adipose |
| 4&14 | Lung (Exhale) |
| 5&7 | $H_2O$ |
| 6&17 | Muscle |
| 8&11 | Trabecular Bone |
| 10&15 | Lung (Inhale) |
| 12&16 | Dense Bone |

**Supplementary Fig. 12** The detail of a thorax phantom. **a,** Morphology of a thorax phantom with movable plugs. Scale bar: 5 cm. **b**, The corresponding organs.

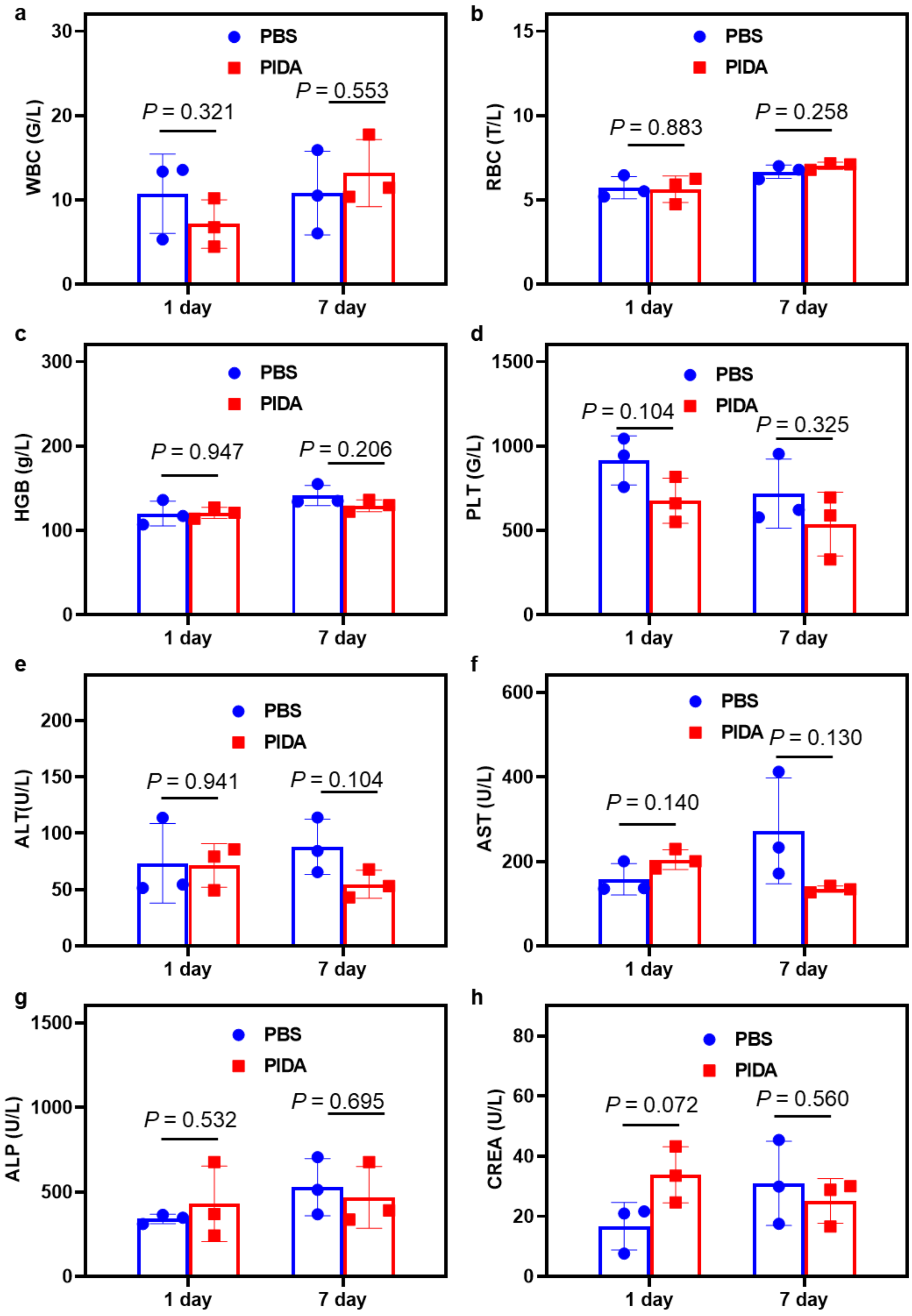


**Supplementary Fig. 13** Blood analysis and liver/kidney function tests of PIDA-injected rats and normal rats (n = 3 biologically independent samples). Data are presented as Mean ± SD. **a**

WBC: white blood cell. **b** RBC: red blood cell. **c** HGB: hemoglobin. **d** PLT: platelet. **e** ALT: alanine aminotransferase. **f** AST: aspartate aminotransferase, **g** ALP: alkaline phosphatase. **h** CERA: creatinine. No significant difference was observed in all samples ($P > 0.05$). One-way analysis of variance was used for multiple-group analysis. Source data are provided as a Source Data file.

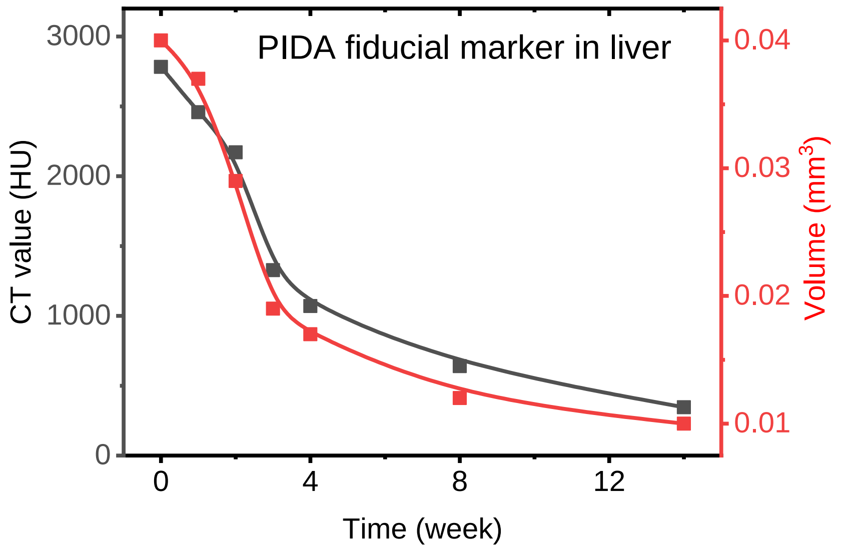


**Supplementary Fig. 14** The changes of the CT value and the volume of the PIDA fiducial marker implanted in rat liver over time. Source data are provided as a Source Data file.

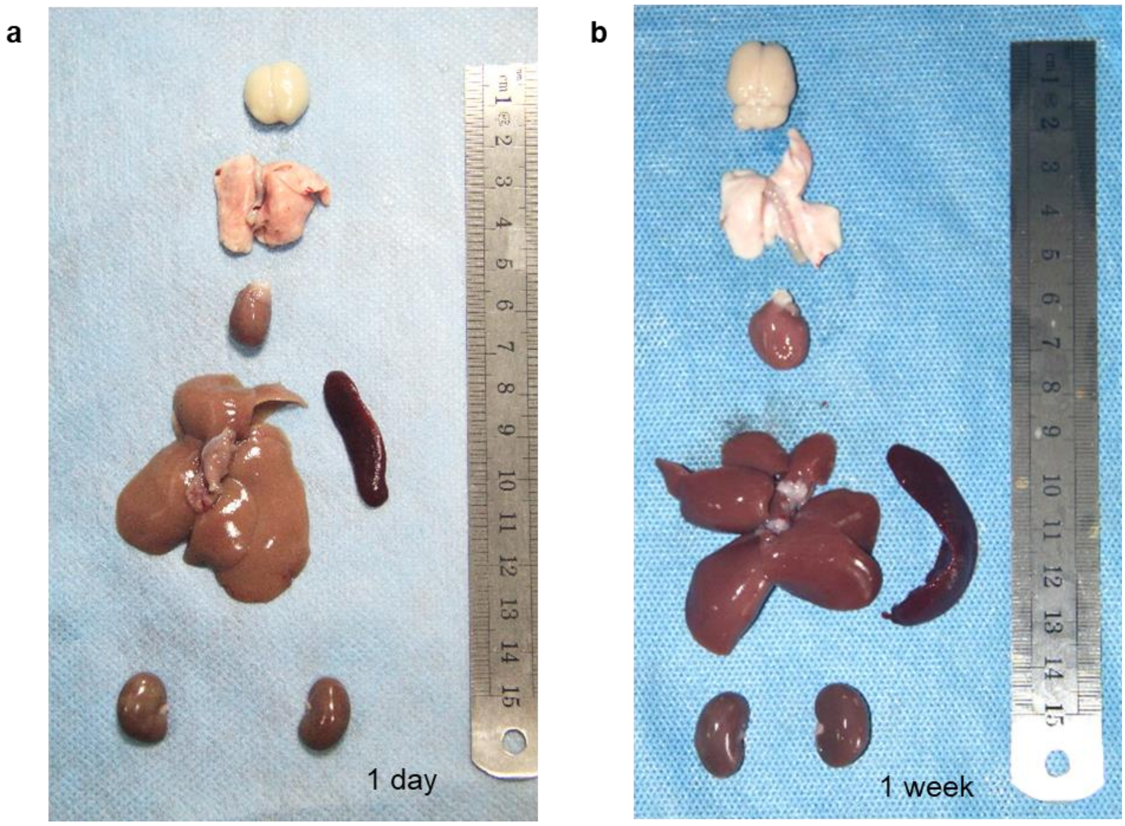


**Supplementary Fig. 15** Photographs of the dissected major organs at 1 day (**a**) and 1 week (**b**) after injected with PIDA. No apparent damage was found in the brain, lung, heart, liver, spleen and kidney.

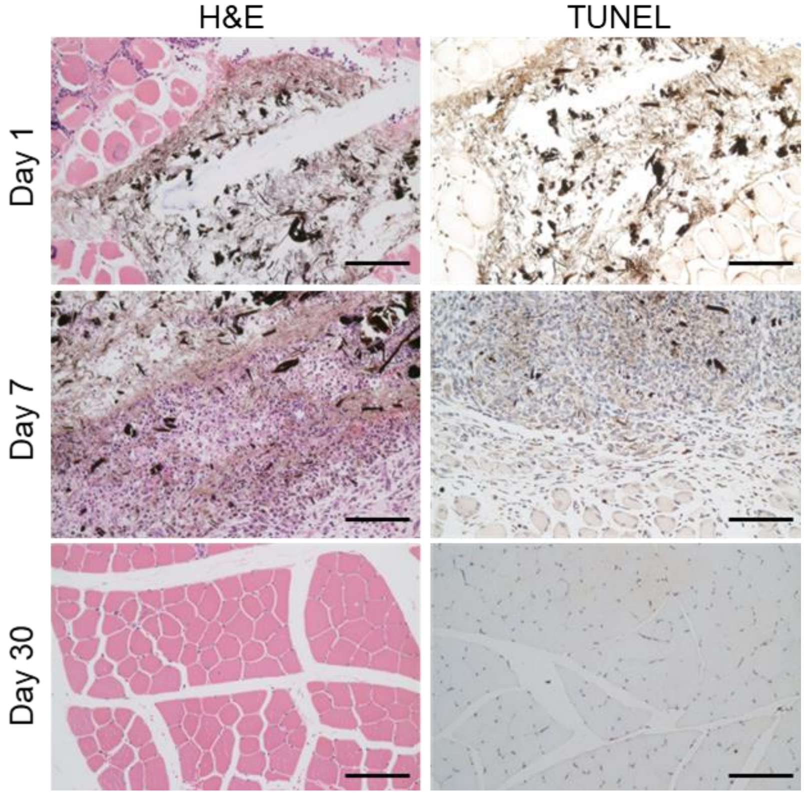


**Supplementary Fig. 16** H&E and TUNEL staining images of the tissues at the injection site of PIDA. Scale bars: 100 μm. The experiment was carried out on biologically independent samples.
