# Supplementary material for: Precisely translating computed tomography diagnosis accuracy into therapeutic intervention by a carbon-iodine conjugated polymer
Source: Nat Commun. 2022 May 12;13:2625. doi: 10.1038/s41467-022-30263-1 (PMC9098856; doi:10.1038/s41467-022-30263-1)
Supplement: Supplementary file 3 — Description of Additional Supplementary Information [file 41467_2022_30263_MOESM3_ESM.pdf]

Inventory of Supporting Information

1. Author Checklist
2. Final article with changes highlighted
3. Reporting Summary
4. Editorial Policy Checklist
5. Supplementary Information.pdf
6. Fig. 1a ChemDraw
7. Fig. 1b ChemDraw
8. Supplementary Fig. 1 ChemDraw
9. Source Data.xlsx
