# Supplementary material for: Precisely translating computed tomography diagnosis accuracy into therapeutic intervention by a carbon-iodine conjugated polymer
Source: Nat Commun. 2022 May 12;13:2625. doi: 10.1038/s41467-022-30263-1 (PMC9098856; doi:10.1038/s41467-022-30263-1)
Supplement: Supplementary file 4 — Reporting Summary [file 41467_2022_30263_MOESM4_ESM.pdf]

nature portfolio

Corresponding author(s): Liang Luo
Last updated by author(s): Apr 7, 2022

Reporting Summary

Nature Portfolio wishes to improve the reproducibility of the work that we publish. This form provides structure for consistency and transparency in reporting. For further information on Nature Portfolio policies, see our Editorial Policies and the Editorial Policy Checklist.

Statistics

For all statistical analyses, confirm that the following items are present in the figure legend, table legend, main text, or Methods section.

n/a	Confirmed	
☐	☒	The exact sample size (*n*) for each experimental group/condition, given as a discrete number and unit of measurement
☐	☒	A statement on whether measurements were taken from distinct samples or whether the same sample was measured repeatedly
☐	☒	The statistical test(s) used AND whether they are one- or two-sided<br>*Only common tests should be described solely by name; describe more complex techniques in the Methods section.*
☒	☐	A description of all covariates tested
☐	☒	A description of any assumptions or corrections, such as tests of normality and adjustment for multiple comparisons
☐	☒	A full description of the statistical parameters including central tendency (e.g. means) or other basic estimates (e.g. regression coefficient) AND variation (e.g. standard deviation) or associated estimates of uncertainty (e.g. confidence intervals)
☐	☒	For null hypothesis testing, the test statistic (e.g. *F*, *t*, *r*) with confidence intervals, effect sizes, degrees of freedom and *P* value noted<br>*Give P values as exact values whenever suitable.*
☒	☐	For Bayesian analysis, information on the choice of priors and Markov chain Monte Carlo settings
☒	☐	For hierarchical and complex designs, identification of the appropriate level for tests and full reporting of outcomes
☒	☐	Estimates of effect sizes (e.g. Cohen's *d*, Pearson's *r*), indicating how they were calculated

Our web collection on statistics for biologists contains articles on many of the points above.

Software and code

Policy information about availability of computer code

Data collection	Data was collected using Nova NanoSEM 450, XtaLAB PRO MM007HF C Single Crystal X ray Diffractometer, TU 1810DSPC UV/Vis spectrophotometer, Hitachi HT7700 for TEM, Agilent 400-MR DDR2 for NMR spectra, TEPMA 8050G for elemental analysis, NGSLabSpec and S&I Vista Control Basic for Raman spectra, SOMATOM Force for CT and Accuray Inc for SBRT.
Data analysis	Statistical analyses were performed using Origin (v. 2018) and GraphPad Prism(v.8.0.2). MestRenNova was used to process NMR spectra (v.6.1.0-6224). Sygno Via was used as a post-processing workstation for CT. The relative CT intensities were derived from corresponding CT images using ImageJ software (v.fiji-win64). The radiation dose calculations were conducted in the same radiotherapy planning using the Halcyon™ Radiotherapy system.

For manuscripts utilizing custom algorithms or software that are central to the research but not yet described in published literature, software must be made available to editors and reviewers. We strongly encourage code deposition in a community repository (e.g. GitHub). See the Nature Portfolio guidelines for submitting code & software for further information.

Data

Policy information about availability of data

All manuscripts must include a data availability statement. This statement should provide the following information, where applicable:

- Accession codes, unique identifiers, or web links for publicly available datasets
- A description of any restrictions on data availability
- For clinical datasets or third party data, please ensure that the statement adheres to our policy

All relevant data are available within the Article, Supplementary Information or Source Data file. The imaging data are available from the corresponding author upon request.

Field-specific reporting

Please select the one below that is the best fit for your research. If you are not sure, read the appropriate sections before making your selection.

☒ Life sciences ☐ Behavioural & social sciences ☐ Ecological, evolutionary & environmental sciences

For a reference copy of the document with all sections, see nature.com/documents/nr-reporting-summary-flat.pdf

Life sciences study design

All studies must disclose on these points even when the disclosure is negative.

Sample size	Details regarding the sample size of all experiments are provided in figure legends. The sample size were consistent with that of previously reported results in other studies.
Data exclusions	No data were excluded from the analyses.
Replication	In vitro experiments were completed in duplicate or triplicate to successfully verify reproducibility. All attempts at replication were successful for the in vitro experiments. In vivo experiments were guided by the relevant doctors. Except for the Beagle experiment, the experiments in vivo shown were performed independently for 3 or more times with success.
Randomization	Samples were randomly allocated to corresponding experimental groups.
Blinding	The investigators were blinded to group allocation during data collection and/or analysis for experiments.

Reporting for specific materials, systems and methods

We require information from authors about some types of materials, experimental systems and methods used in many studies. Here, indicate whether each material, system or method listed is relevant to your study. If you are not sure if a list item applies to your research, read the appropriate section before selecting a response.

Materials & experimental systems

n/a	Involved in the study	
☒	☐	Antibodies
☐	☒	Eukaryotic cell lines
☒	☐	Palaeontology and archaeology
☐	☒	Animals and other organisms
☒	☐	Human research participants
☒	☐	Clinical data
☒	☐	Dual use research of concern

Methods

n/a	Involved in the study	
☒	☐	ChIP-seq
☒	☐	Flow cytometry
☒	☐	MRI-based neuroimaging

Eukaryotic cell lines

Policy information about cell lines

Cell line source(s)	The metastatic murine 4T1 breast cancer cells, the fibroblast NIH 3T3 cells, the HEK 293T human embryonic kidney cells were kindly provided by Chenhui Wang, Huazhong University of Science and Technology. The Walker 256 malignant ascite cells were kindly provided by Bin Xiong, Huazhong University of Science and Technology.
Authentication	The cell lines were morphologically confirmed according to the information provided by ATCC
Mycoplasma contamination	The cell lines were not tested for mycoplasma contamination.
Commonly misidentified lines (See ICLAC register)	This study did not involve misidentified cell lines.

Animals and other organisms

Policy information about studies involving animals; ARRIVE guidelines recommended for reporting animal research

Laboratory animals	SD male rats (6–8 weeks old, 160-180 g) and the male Beagle (1 year old, 8 Kg) were acquired from the Laboratory Animal Centre of Hubei, China.
Wild animals	This study did not involve wild animals.

Field-collected samples	This study did not involve field-collected samples.
Ethics oversight	Animal protocols were performed under the guidelines for human and responsible use of animals in research set by Institutional Animal Care and Use Committee, Huazhong University of Science and Technology ([2021] IACUC Number: 2622).

Note that full information on the approval of the study protocol must also be provided in the manuscript.
